# Author Correction: Development of a novel risk score to predict mortality in patients admitted to hospital with COVID-19

**DOI:** 10.1038/s41598-021-87439-w

**Published:** 2021-04-07

**Authors:** Ying X. Gue, Maria Tennyson, Jovia Gao, Shuhui Ren, Rahim Kanji, Diana A. Gorog

**Affiliations:** 1 University of Hertfordshire, College Lane, Hatfield, AL10 9 AB UK; 2 East and North Hertfordshire NHS Trust, Lister Hospital, Stevenage, Hertfordshire SG1 4AB UK; 3 National Heart and Lung Institute, Imperial College, Dovehouse Street, London, SW3 6LY UK

Correction to: *Scientific Reports* https://doi.org/10.1038/s41598-020-78505-w, published online 07 December 2020

The original version of this Article contained an error, where the values for “sensitivity” and “specificity” were switched.

As a result, in the Abstract,

“The optimal cut-point was a score ≥ 4, which had a sensitivity of 78.36% and a specificity of 67.59%.”

now reads:

“The optimal cut-point was a score ≥ 4, which had a specificity of 78.36% and a sensitivity of 67.59%.”

In addition, in the Results section, under the subheading ‘Development of a COVID-19 mortality risk score’,

“The optimal cut-point with the highest combined sensitivity and specificity was a score of ≥ 4, which has a sensitivity of 78.36% and a specificity of 67.59%, with a positive predictive value of 72.6% and negative predictive value of 74%.”

now reads:

“The optimal cut-point with the highest combined sensitivity and specificity was a score of ≥ 4, which has a specificity of 78.36% and a sensitivity of 67.59%, with a positive predictive value of 72.6% and negative predictive value of 74%.”

The original Article has been corrected.

